# Commentary: Optimal left ventricular assist device duration in bridge to transplantation: Do we have a choice now, or is it a moot point?

**DOI:** 10.1016/j.xjon.2021.10.030

**Published:** 2021-10-23

**Authors:** Sandeep Sainathan, Tomas A. Salerno

**Affiliations:** a Division of Congenital Heart Surgery, University of Miami Miller School of Medicine, Miami, Fla; b Division of Cardiothoracic Surgery, University of Miami Miller School of Medicine, Miami, Fla; c Department of Cardiac Surgery Jackson Memorial Hospital, Miami, Fla

**Figure:**
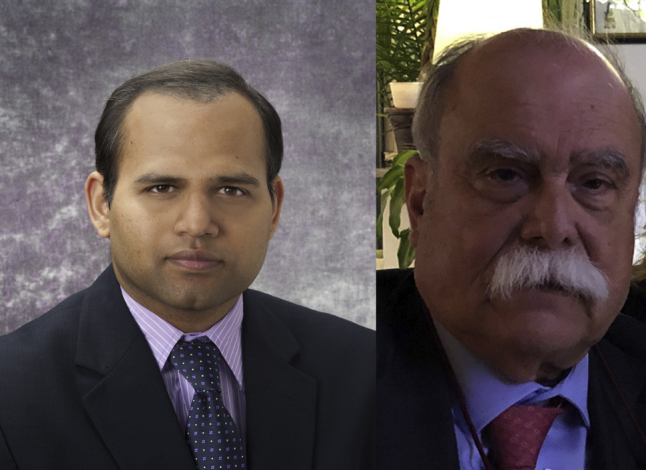
Sandeep Sainathan, MD, and Tomas A. Salerno, MD

**Central Message**

The role of implantable durable LVADs as a bridge to HTX, and timing of transplantation postimplantation, continues to evolve.

See Article page 116.

Goodwin and colleagues,^1^ in this expert review, describe the optimal time frame to perform heart transplantation (HTX) in patients who are being bridged with an implantable durable left ventricular assist device (iLVAD). As pointed out by the authors, HTX in patients before 30 days and/or after 9 months of iLVAD implantation may be associated with suboptimal outcomes. This is likely due to insufficient recuperation time, or early device-related complications when HTX happens within the first 30 days, or late device complications when HTX occurs later than 9 months of iLVAD implantation. They suggest that a period of 1 to 3 months post-iLVAD implantation is the optimal window for HTX, although this can be extended to 6 months if spontaneous cardiac recovery is a possibility. This review comes at a time when there have been significant changes in the US heart allocation system and the current state of development of iLVAD devices. Many of the conclusions outlined in their report are based on data that preceded these changes. Thus, the optimal timing for HTX in iLVAD patients may not be achievable or, alternatively, be a moot point.

With the new 6-tier HTX listing system put into effect October 2018, priority for patients supported with an iLVAD has been moved down the list to Status 4, by default.^2^ Also, allocation of local donor hearts to eligible recipients, up to 500 nautical miles, has further increased competition for hearts. This has led to increased waitlist times for these patients.^2^ The goal of iLVAD implantation was to avoid further end-organ injury and resuscitate patients with medically refractory heart failure before proceeding to HTX. With the newer listing system, temporary mechanical circulatory support devices have assumed much of this role, as they provide higher listing status and, thus, shorter waitlist times.^3^ Thus, current role of iLVAD in context of bridge to HTX is under re-evaluation. It may be suitable for patients who have exhausted high priority for HTX on temporary mechanical support device, who have high pulmonary vascular resistance, may be amenable to remodeling with an iLVAD, or are in need of desensitization therapy. With longer intent of support and lower priority on HTX list, optimal timing may not be achievable, as suggested by these authors in this comprehensive review.

HeartMate 3 (Abbott Cardiovascular, Chicago, Ill) is currently the only available iLVAD in the US market. Outcomes of this device have been superior to any previous devices, particularly regarding pump thrombosis and stroke. Results of 2-year survival in patients receiving this device, as destination therapy, have been similar to survival with HTX. This suggests that decision of either current or future HTX eligibility should not be made apriori.^4^ Thus, the effect of more extended device support on morbidities, such as pump thrombosis and stroke, may be less with this device. Therefore, urgency to HTX may be a moot point. With this device's built-in pulsatility mechanism, previous concerns for the development of post-transplantation vasoplegia, due to prolonged device support, need to be further evaluated.
